# Nutritional Characteristics of Prepacked Feta PDO Cheese Products in Greece: Assessment of Dietary Intakes and Nutritional Profiles

**DOI:** 10.3390/foods9030253

**Published:** 2020-02-27

**Authors:** Evangelia Katsouri, Emmanuella Magriplis, Antonios Zampelas, George-John Nychas, Eleftherios H. Drosinos

**Affiliations:** 1 Hellenic Food Authority, 11526 Athens, Greece; ekatsouri@efet.gr; 2 Department of Food Science and Human Nutrition, School of Food and Nutritional Sciences, Agricultural University of Athens, 11855 Athens, Greece; emagriplis@aua.gr (E.M.); azampelas@aua.gr (A.Z.); gjn@aua.gr (G.-J.N.)

**Keywords:** feta cheese, dairy products, nutritional characteristics, nutrient intake, nutrient profile, nutrition claims, nutritional labeling

## Abstract

Feta cheese, a protected designation of origin (PDO) food, is one of the most important Mediterranean food products. Although it is the cheese with the highest consumption in Greece, the nutritional characteristics of products available in the market, as well as their contribution to the Greek diet, have not been evaluated in detail. In the present study, the basic nutritional content of 81 prepacked feta cheese products available in the Greek market were recorded based on their labels. This was combined with consumption data to provide an overall picture of feta cheese’s contribution to the Greek diet. The nutrient contents per 100 g ranged as follows. Energy: 221–343 kcal, total fat: 20–29 g, saturated fat: 12.8–20.3 g, carbohydrates: 0–3.1 g, sugars: 0–3 g, proteins: 13.1–21.0 g and salt: 1.2–5.1 g. The median feta daily individual consumption was found to be 39 g, ranging from 20 g to 100 g (fifth and 95th percentiles, respectively). The nutritional intake analysis as a percentage of dietary reference intake (DRI) showed that saturated fat and salt are ranked on the top of the list, with intakes reaching 101.5% and 85% respectively. The products were also evaluated against five nutrient profile models and their potential use under statutory requirements and policy development are discussed.

## 1. Introduction

Feta cheese has been produced since Homer’s time. It is the best known Greek cheese, with a prominent place in the Greek and international market, and it is ranked first in export sales [1]. Since 2002, feta has been a protected designation of origin (PDO) product in the European Union [2]. According to the relevant EU legislation, PDO foodstuffs must comply with certain specifications related to name, raw material origin and characteristics, description of production method, definition of the geographical region of origin and production, details for the inspection structures and specific labeling details [3]. Feta PDO cheese, specifically, must be produced from sheep’s milk, or from a mixture of sheep’s and up to 30% goat’s milk in particular areas of Greece (Macedonia, Thrace, Epirus, Thessaly, mainland Greece, Peloponnesus, Lesvos, Limnos, Agios Efstratios). Most feta cheese is produced from pasteurized milk in organized cheese dairy establishments, using commercial lactic acid cultures [4]. Production includes an acidification step aided by the addition of yoghurt starter cultures containing *Streptococcus thermophilus* and *Lactobacillus delbrueckii* subspecies *bulgaricus*. The fresh cheese is dry-salted for 4–5 days, is then placed in brine with an approximately 8% salt content for ripening for at least 60 days, and is later commercialized [5]. Feta cheese is traditionally distributed in metal vessels or wooden barrels and sold through the retailers’ service counters. Recently, however, prepacked feta cheese products sold at super market shelves have gained a significant share in the Greek and international market. The fact that in the latter case consumers have an immediate access to the product label raised the interest of both the dairy industry and the consumers to the nutritional characteristics of the product as well as the potential nutritional claims that could be included in the label.

Composition and nutrient characteristics of feta cheese depend on an increased number of factors including the composition of raw material (milk), microbial ecology of the product, salt content, duration and conditions of ripening and others. For example, the salting method, which can vary among producers, can significantly affect both the salt and the fat content of the final product. On the other hand, ripening conditions and time affects the final cheese composition, since these factors determine the type and extent of lipolysis and proteolysis [6,7].

Despite the importance of feta cheese in Greece, data on the nutritional characteristics of the different products marketed in Greece as well as on their contribution to the daily nutrient intake for the Greek population are limited. Such data however, are very important for the development of healthy diet strategies. For example, a recent survey on salt intake in Greece [8] showed that only 5.6% of consumers had a salt intake of less than 5 g/day, which is the target intake recommended by the World Health Organization [9], while 50.4% of consumers had a daily salt intake that exceeded 10 g per day. Considering the high consumption of feta in Greece and the fact that it is a product with a high salt concentration an evaluation of its contribution to the daily salt (and other nutrient) intake, is of great importance in order to develop effective salt reduction strategies. The importance of such strategies is supported by actions in the European Union that encompass salt monitoring and evaluation of salt reduction actions, as one of their important pillars [10].

Additionally, many consumers around the world are increasingly focused on healthy eating and many actively make dietary choices to reduce risk of various health issues such as obesity, diabetes, high cholesterol and hypertension [11]. A comparative study of 12 food values between the United States and Norway using the best–worst scaling approach showed that respondents in both countries have similar food values, with safety being scaled as the most important value and nutrition ranked 6th [12]. Moreover, various studies on consumers’ “willingness to pay” (WTP) have reported that PDO regional products are highly appreciated [13,14,15] and are perceived by consumers as healthier [16]. Other studies have reported that consumers expect that products with nutrition and health claims on the packaging have a better overall nutritional value compared with products without such information [17,18]. Taking these reports into account one must also consider that consumers may be biased and confused from labeling information [19], hence providing nutritional and health information to consumers in an effective way, remains a challenge for the food industry.

Food labels are the main method for transferring nutrition and health information of foodstuff to consumers [19]. In Europe, information made on food is regulated by specific laws, including (i) the European Regulation (EU) no 1169/2011, which regulates the mandatory information on food including ingredient lists and nutritional declarations [20]; and (ii) the European Regulation (CE) no 1924/2006, concerning the voluntary nutrition and health claims (NHC) [21]. According to Reg. (EC) 1924/2006, Article 4, “*the Commission shall establish specific nutrient profiles and the conditions, including exemptions, which shall be respected for the use of nutrition and health claims on foods and/or categories of foods*”. Nutrient profiling involves the classification and ranking of foods according to their nutritional composition for reasons related to preventing disease and promoting health [22]. However, the setting of nutrient profiles has been postponed, due to the complexity of the subsequent discussions in relation to scientific issues and potential economic effects. Nevertheless, various optional nutrient profile models (NPMs) have been developed in several countries based on conditions regulated by their particular population and needs [23]. The evaluation of feta cheese products against available NPMs would provide the basis for the Greek dairy industry to establish the nutrient profile and to prepare future setting of nutrition or health claims in feta cheese.

The present study aimed to evaluate all previously mentioned nutritional aspects of prepacked feta PDO cheese in Greece and assess percent contribution of feta to salt and saturated fat intake of a representative population sample to recommended intakes. Specifically, the main objectives were (a) to comparatively assess the nutritional characteristics of prepacked PDO feta cheese products available in the Greek market, (b) to combine the nutritional characteristics with consumption data in Greece in order to evaluate the contribution of feta cheese consumption to the Greek diet compared to the European daily reference intake (RI) values and (c) to evaluate the nutritional characteristics of feta cheese products against available NPMs, providing evidence on nutritional profile and future setting of nutritional or health claims in feta cheese.

## 2. Materials and Methods

### 2.1. Sampling, Data Collection and Analysis of Nutritional Characteristics of Prepacked Feta Cheese

Sampling of prepacked feta cheese products took place in supermarkets, discount and cash & carry chain stores of all major retailers (Lidl, AB-Delhaize, Sklavenitis, Masoutis, Elomas, Kritikos, My market, Market In, Discount Markt, Mako Markets, Spar, A/S Agora, Galaxias, Makro, The Mart) in three Greek cities (Athens, Thessaloniki, Larisa) during September-December 2018. In total, 81 feta PDO cheese products, produced by 55 feta manufacturers, were identified and sampled. All sampled products were purchased and photographed, and their packages were retained. For each product all nutrients available on the labeling nutrition declaration were retrieved separately. Data, including all labelling information were retrieved from the images of all the sides of each product-package sampled. More specifically, all nutrients available on the labeling nutrition declaration: energy (kcal), protein (g), carbohydrates (g), total sugars (g), fat (g), saturated fat (g), and salt (g) per 100 g were retrieved separately and were analyzed statistically. This information was entered in a specially created database along with a photo of the product. The database was used as a data depot for further statistical analysis.

### 2.2. Analysis of Nutrient Intake by Feta Cheese Consumption

Nutrient intakes of healthy Greek adults from feta cheese consumption were calculated per capita and per day, using the nutrient contents of the 81 sampled products in combination with feta cheese consumption data obtained from the Hellenic National Nutrition and Health Survey (HNNHS). Specific study details have been published [24]. To evaluate the daily consumption per capita of feta cheese in Greece, consumption data from 1232 adults (46.5% males) from the HNNHS who had declared to consume feta cheese were used. In order to describe nutrient intake variability, feta cheese consumption, median and range were calculated (fifth, 50th and 95th percentiles) based on daily per capita consumption and the mean nutrient content of the 81 tested products. The intake of nutrients was also expressed as percentage of the European daily reference intake (DRI) values as set by the European legislation [20]. The RI values used were energy: 2000 kcal, total fat: 70 g, saturated fat: 20 g, carbohydrates: 260 g, sugars: 90 g, proteins: 50 g and salt: 6 g.

### 2.3. Evaluation of the Nutritional Characteristics of Feta Cheese Products against Available Nutritional Profiling Models (npms)

The 81 prepacked feta cheese products identified in the Greek market were evaluated against the following five NPMs. Model I: The World Health Organization Nutrient Profile Model (WHO-NPM), model II: The Swedish Keahole (SK-NPM), model III: The United Kingdom Nutrient Profile Model (UK-NPM), model IV: The Food Standards Australia New Zealand Nutrient Profile Scoring Criterion (FSANZ NPSC) and model V: The Choices Programme (CP-NMP).

Models (I), (II), (V) are threshold models while III and IV are scoring models. Model I [25] is a threshold model which sets criteria on two basic nutrients (total fat and salt), aiming to inform product policy development directed to children. Model II [26] is a threshold model which sets criteria on two basic nutrients (total fat and salt) with scope to qualify the products for related health claims. Model III [27] is a scoring model developed to regulate food marketed to children and attempts to balance the contribution from “beneficial” nutrients of food alongside the “negative”. Model IV [28] is a scoring model which categorizes food based on specific characteristics (e.g., for cheese: calcium content). Model V [29] is a threshold model which sets criteria on the three basic nutrients (saturated fat, salt and no added sugars) with scope to qualify products for health claim use. All the above NPMs have been developed by government, global, or other agencies and have been used to categorize products according to their nutritional characteristics [17,30,31]. A detailed description of the selected NPMs is presented in Table 1.

The evaluation of the prepacked feta cheese products against the NPMs was based on their nutrient contents recorded in the first part of the study. For model I, it was not taken into account, that, according to the model’s terms, “if the product is a food that has a protected designation of origin or a protected geographical indication or is a guaranteed traditional specialty, marketing (to children) may be permitted according to national context” [25]. For model IV, estimations were made with the assumptions of a 39 g serving and an average Ca content of 0.450 g/100 g for all products based on literature data [32,33]. The assumed serving of 39 g for feta cheese corresponds to the median value of feta consumption according to HNNHS data.

## 3. Results

### 3.1. Nutritional Characteristics of Prepacked Feta Cheese

**Figure 1:**
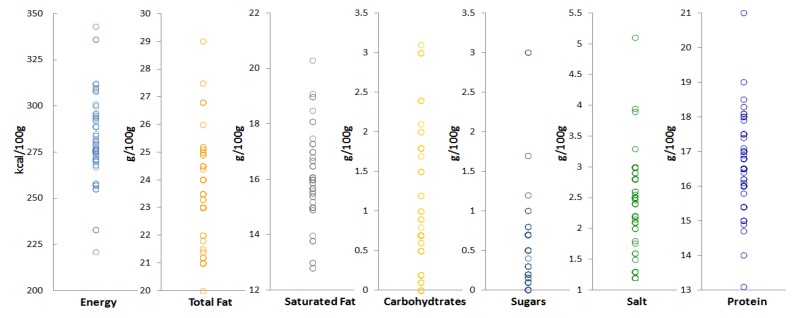
Distributions of energy, total fat, saturated fat, carbohydrates, sugars, salt and protein per 100 g, for prepacked feta cheese products available in the Greek market.

In total, 81 products of prepacked feta cheese were identified in the major Greek retail chains produced by 55 dairy companies. According to their labeling information and production’s establishment approval number the majority of the products (72.9%) were produced in approved production establishments [34] in four of the nine PDO qualified administrative districts [Thessaly (21%), Central Macedonia (19.8%), Epirus (17.3%) and West Greece (14.8%) of PDO. The distributions of the nutrient contents (energy, protein, carbohydrates, total sugars, fat, saturated fat, and salt per 100 g) of the products according to their nutrition declaration are presented in Figure 1. Table 2 presents the descriptive statistics of the nutrient content of the 81 products.

The results showed that there is a significant variation in the nutrient content of feta cheese products. In particular, the observed ranges per 100 g were energy: 221–343 kcal, total fat: 20–29 g, saturated fat: 12.8–20.3 g, carbohydrates: 0–3.1 g, sugars: 0–3 g, proteins: 13.1–21.0 g and salt: 1.2–5.1 g. The coefficient of variation (%CV = SD/mean * 100) for the different nutrients ranged from 6.8% for total fat to 120% for sugar.

### 3.2. Nutrient Intake by Prepacked Feta Cheese Consumption in Greece and Comparison with the Respective European DRIs

Feta cheese consumption data for 1232 healthy adult Greek consumers who had declared to consume feta cheese, were obtained from the Hellenic National Nutrition and Health Survey database [24] and analyzed. The descriptive statistics of the consumption data are shown in Table 3.

**Figure 2:**
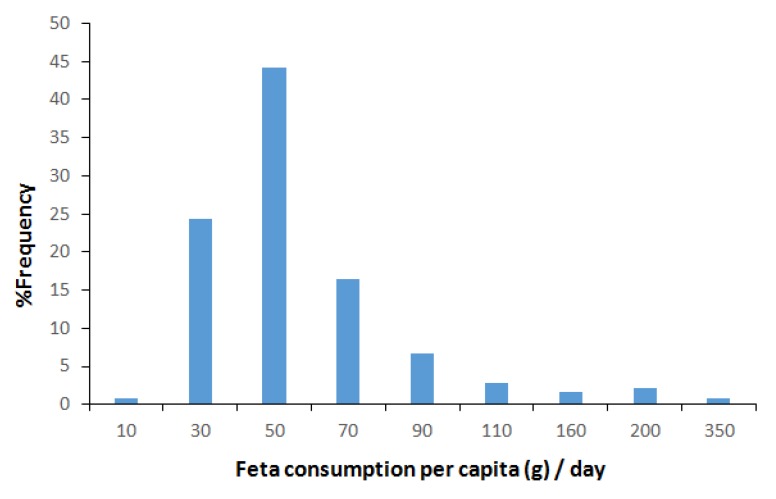
Frequency histogram of Greek adults’ feta cheese consumption (g) per capita and per day based on data 1232 healthy adult Greek consumers extracted from the Hellenic National Nutrition & Health Survey (HNNHS) database.

In Figure 2, the frequency histogram of Greek adults’ feta cheese consumption (g) per capita and per day, is shown.

The results from the analysis of the consumption data showed that feta cheese consumption varied significantly among Greek consumers. Consumption per capita per day ranged from 5 g to 336 g with an average value of 50.3 g and a median value of 39 g. The estimated %CV was 92.8% and the distance between the fifth percentile (20 g) and the 95th percentile (100 g) was 80 g. The data on feta cheese consumption per capita per day were combined with the data on the basic nutrients’ content of the prepacked feta cheese products available in the Greek market in order to provide an overall picture of feta cheese contribution to the Greek diet. In order to describe the variability of both daily consumption and nutrient’s content among the various products the fifth, 50th and 95th percentiles were used. Table 4 presents the daily intake of feta basic nutrients based on the fifth, 50th and 95th percentiles of nutrient contents in the product and daily consumption of feta according to HNNHS data.

**Figure 3:**
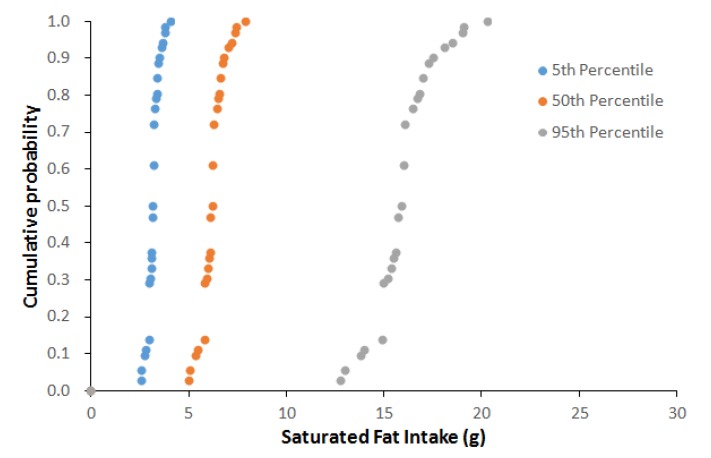
Cumulative probability of saturated fat (g) intake per capita and per day of Greek adults’ consuming feta cheese marketed in the Greek market for the fifth, 50th and 95th percentiles of daily consumption.

**Figure 4:**
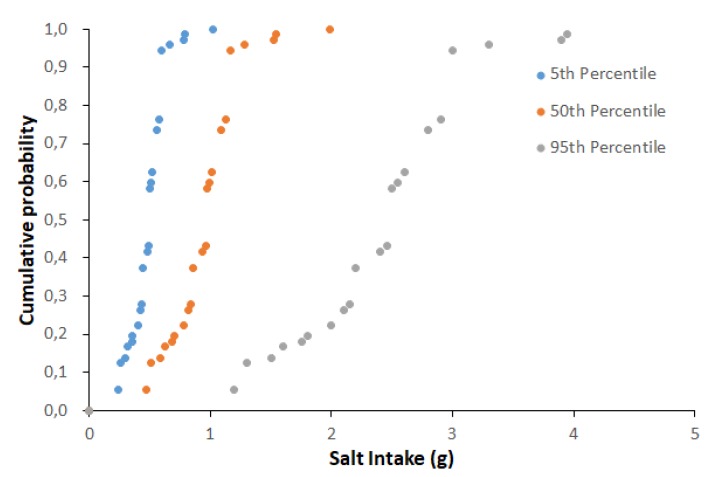
Cumulative probability of salt (g) intake per capita and per day of Greek adults’ consuming feta cheese marketed in the Greek market for the fifth, 50th and 95th percentiles of daily consumption.

Table 4 provides and overall picture of the variability in nutrient intake by consumers of prepacked feta cheese in Greece originated from the differences in nutrient content among products available in the market and the daily consumption quantity among consumers. For example, the salt daily intake for the 50th percentile of feta daily consumption and the 50th percentile of salt content in prepacked feta, representing a scenario of an average consumer eating a product with an average salt concentration, was estimated to 1 g. For an adult consuming prepacked feta cheese at the highest quantity range (95th percentile) of a product with the higher salt concentration among those available in the market (95th percentile), the salt daily intake increases significantly to 3.3 g, from feta cheese alone. Representative cumulative probability of saturated fat and salt intake per capita and per day, of Greek adults’ consuming feta cheese marketed in the Greek market for the fifth, 50th and 95th percentiles of daily consumption also presented in Figure 3 and Figure 4.

Nutrient intake was expressed as percentage of the European daily reference intake (DRI) values as set by the European Regulation (EU) 1169/2011 on the provision of food information to consumers, in order to demonstrate the contribution of feta cheese consumption to a healthy adult’s diet.

**Figure 5:**
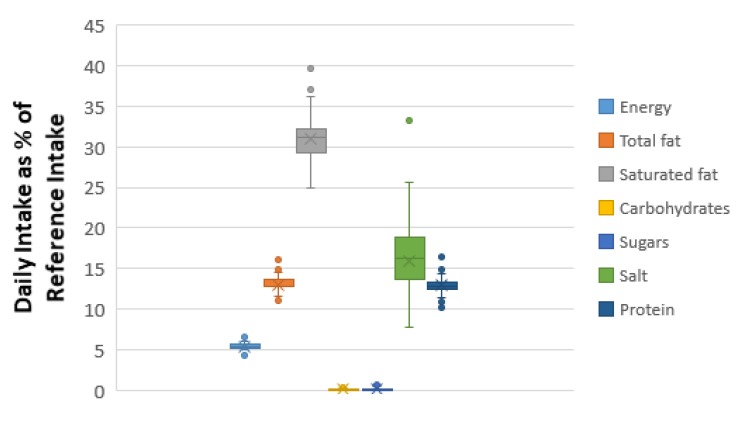
Daily intake per capita as a percentage of European daily reference intakes (RIs), for the 50th percentile of the daily consumption of prepacked feta cheese marketed in the Greek market.

**Figure 6:**
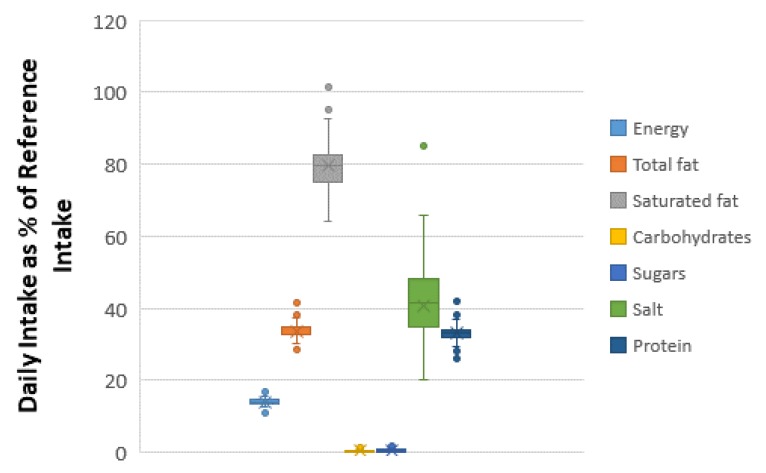
Daily intake per capita as a percentage of European daily reference intakes (RIs), for the 95th percentile of the daily consumption of prepacked feta cheese marketed in the Greek Market.

Figure 5 and Figure 6 present the box plots of the daily nutrient intake as DRI percentage by feta cheese consumption for the 50th and 95th percentiles of daily consumption quantity. For the 50th percentile of daily feta consumption (corresponding to 39 g), the estimated ranges for energy, total fat, saturated fat carbohydrates, sugars, proteins and salt were 4.3–6.7%, 11.1–16.2%, 25–39.6%, 0–0.5%, 0–1.3%, 10.2–16.4% and 7.8–33.2%, respectively. For the 95th percentile of daily feta consumption (corresponding to 100 g), the %RI for energy, total fat, saturated fat carbohydrates, sugars, proteins and salt were 11–17.2%, 28.5–41.4%, 64–101.5%, 0–1.2%, 0–3.3%, 26.2–42% and 20–85%, respectively.

### 3.3. Evaluation of the Nutritional Profile of Feta Cheese Products against Five NPMs

The 81 prepacked feta cheese products were evaluated against three threshold (I, II, and V) and two scoring (III and IV) nutrient profile models. The results of the evaluation are presented in Table 5.

The results showed that almost all products failed the criteria of models I, II and III. This can be attributed to the high levels of total fat, saturated fat and salt content. A very low number of products (5%) that had a favorable combination of saturated fat and salt content was qualified against model V. Regarding the evaluation against model IV, all products (100%) were qualified based on the assumption that Ca content is 450 mg/100 g and the serving unit of 39 g. However, considering the expected variability in the Ca content among products and the serving unit among consumers, the qualification of a product to the latter model may also vary. For example, for Ca content ≤320 mg/100 g and a 39 g serving unit 0% of the products are qualified, while for the same Ca content and a 25 g serving unit, the percentage of qualified products increased to 30%. Thus, to evaluate the prepacked feta cheese products against model IV, further research is required on the variability of Ca content.

## 4. Discussion

The analysis of the nutritional content of prepacked feta cheese products in the Greek market performed in the first part of the present study, showed that the average values are in agreement with previously reported nutrient content of feta cheese [4]. However, a significant variability in the nutrient content among the products was observed, which can be attributed to the differences in raw material (milk), production methods and conditions among feta cheese producers. Indeed, Pappas et al. [6] manufactured feta cheese by using five different salting methods and reported significant differences in both salt and fat content of the final products. McMahon et al. [35] reported that the salt concentration in brine and the temperature of brining may significantly affect the moisture of feta and thus all nutrients content per 100 g. Moisture content may also be affected by the final pH of feta cheese as well as the ratio of goat’s and sheep’s milk used [35,36]. It needs to be noted that this is the first study providing quantitative data on the variability of nutrient content among feta cheese products available in the Greek market.

In the second part of the present study, feta cheese consumption data were extracted from the HNNHS database and analyzed. The latter consists in an in-depth analysis which characterizes the variability in feta cheese consumption among Greek consumers based on a very large sample (1232 consumers). The results from this analysis showed a high variability of the quantity of feta cheese consumed per capita per day. The estimated median daily consumption of 39 g is nevertheless in accordance with previously published consumption quantities for feta cheese. Specifically Manolopoulou et al. [37], reported that an average annual consumption per capita of this cheese in Greece is approximately 12 kg, which corresponds to a daily consumption of 32.8 g.

Since the available studies connecting nutritional characteristics of food products with contribution to diet and health are very limited [38], the present study attempted to give an overview of the nutritional quality of feta PDO products present on the Greek market with a focus on their differences and their contribution to the Greek diet by combining feta consumption data from the Greek population. The results showed that the estimated daily intake of basic nutrients by feta cheese consumption for a healthy adult varied significantly depending on the consumption quantity and the selection of the product from among these available on the market. The ranking of daily nutrient intake from prepacked feta cheese consumption, estimated as percentage of European RI, was (from higher to lower quantity): 1-saturated fat, 2-salt, 3-total fat, 4-protein, 5-energy, 6-carbohydrates, 7-sugars. Among them, intake of saturated fat and salt may exceed the RI with percentages up to 101.5% and 85% of RI, respectively. These results are supported by recent studies, reporting high salt intakes observed in Greece [8,39]. In particular, 50.4% of the adults in SING study [8] had a daily salt intake which exceeded 10 g per day while WHO recommendations for salt intake limit is 5 g/day, and 23% of children in GRECO study [39] reached high percentages regarding daily salt intake. Given that Greeks consume feta cheese almost on a daily basis, and that its consumption covers the largest part of total domestic consumption [1], feta, may consequently have a significant contribution on saturated fat and salt intake on the Greek population’s diet, as this study showed. This is also supported by the study of Athanasatou et al. [40], who reported that the main contributors to sodium intake in Greece are dairy products (including cheese, yogurt and milk), breads and snacks, in descending order.

The above results confirm the need for the development of strategies for reducing saturated fat and salt intake in Greece, including policy initiatives, industry interventions and improvement of food label information provided to consumers, regarding the nutritional content and healthiness of food. In the policy field, WHO has published a Guideline on salt reduction [9] and launched a public consultation on draft guidelines for intake of saturated fat and trans-fat. The objective of the latter guideline is the reduction of cardiovascular diseases in adults and children through recommendations about saturated fat and trans-fat intakes [41]. Similarly, the European Union encompasses monitoring and evaluation actions as one of their important pillars in reducing salt intake [10]. To the best of our knowledge the Hellenic Food Authority has also launched a strategic plan on the reduction on salt [42]. The data provided in the present study could be helpful for the development of such strategies.

Regarding the Greek dairy industry, possible intervention strategies to reduce salt intake by feta cheese consumption may include nutritional reformulation such as the partial substitution of NaCl by KCl. Indeed, Katsiari et al. [7], reported that feta cheeses made with mixtures of NaCl/KCl exhibited no significant (*p* > 0.05) differences in compositional (moisture, fat, protein, salt), physicochemical (pH, a_w_), sensory (appearance, body and texture, flavor, overall quality) and textural (force and compression to fracture, hardness) properties in comparison with the control cheese. They also showed that the 1:1 NaCl/KCI mixture in the salting of feta cheese effectively brought its Na:K ratio in the final products close to 1 while reducing the sodium content by about 50%. Such a salt reduction can definitely support the use of the comparative nutrition claim “LESS SALT”, which is already being used by industry in feta’s prepacked products, in a small percentage that manages to satisfy the claim’s criteria.

Regarding improvement of food label information provided to consumers, the study supports that the implementation of a selected nutrient profile scheme not only for products bearing NHC but also for products with other type of health-related label information and geographical indications (GI)’s, should be established and be mandatory either in European or national level as also suggested by other studies [17]. Nutrient profiling could serve as a tool for consumers in order to identify products with a high content of “negative” nutrients such as saturated fat and salt and make healthier choices. Nevertheless, attention is needed on too restrictive NPMs that could lead to exclusion or rejection of products with potentially beneficial effects on human diet, due to specific positive nutrients such as calcium, in dairy products, which may not be taken into account in the NPM. Indeed, Trichterborn et al. [43], showed that too restrictive nutrient profile models could help reducing the intake of salt and saturated fat of dairy products but could also negatively impact the intake of calcium and vitamin D. The latter is confirmed by the results of the present study, which showed that feta cheese products could be qualified only against NPM’s which take into account the Ca content. Feta ‘s Ca content in addition, can evidently support the use of “source of calcium” or “high in calcium” nutrition claims, but analysis is needed on a case-by-case basis.

PDO food products such as feta cheese, already highly appreciated by consumers, need to point out their historical and nutritional quality by complying with legislation and making accurate use of available tools. Future setting of nutrition claims and the possibility of a potential inclusion of minimum nutritional requirements in PDO specifications could possibly be examined. The results of the present study provide feta PDO cheese with useful data on these directions.

## Figures and Tables

**Table 1:** Overview of the five chosen nutrient profile models and their key parameters.

Model Number	Model Name	Calculation Approach	Reference Quantity	Number of Nutrients (Negative/Positive)	Nutrients to Limit (Negative)	Nutrients to Encourage (Positive)	Reference
I	World Health Organization Nutrient Profile Model (WHO-NPM)	THRESHOLD	100 g	7	total fat 20 g/100 g, salt 1.3 g/100 g	-	[25]
II	Swedish Keyhole (SK-NPM)	THRESHOLD	100 g	(5/1)	total fat 17 g/100 g, salt 1.6 g/100 g	fiber-wholegrain	[26]
III	United Kingdom Nutrient Profile Model (UK-NPM)	SCORING	100 g	(4/3)	energy, saturated fat, total sugar, sodium	fruits, vegetables and nuts, fiber, protein	[27]
IV	Food Standards Australia New Zealand Nutrient Profile Scoring Criterion (FSANZ NPSC)	SCORING	one serving	(4/3)	energy, saturated fat, total sugar, sodium	fruits, vegetables and nuts, fiber, protein	[28]
V	Choices Programme Nutrient Profile Model (CP-NMP)	THRESHOLD	100 g	(4/1)	total fat 15 g/100 g, trans fatty acids 0.1 g/100 g, sodium 8.3 g/100 g, salt 2.075 g/100 g, no added sugars	fiber	[29]

**Table 2:** Descriptive statistics of nutritional characteristics (per 100 g) of prepacked protected designation of origin (PDO) feta cheese products in the Greek market.

	Energy (kcal)	Total Fat (g)	Sat. Fat (g)	Carbohydrates (g)	Sugars (g)	Proteins (g)	Salt (g)
**Mean**	280.5	23.4	15.9	0.9	0.5	16.6	2.4
**St. Error**	2.3	0.2	0.2	0.1	0.1	0.1	0.1
**Median**	276.0	23.0	16.0	0.7	0.5	16.5	2.5
**Mode**	276.0	23.0	15.0	0.7	0.7	16.5	3.0
**St. Dev.**	20.3	1.6	1.4	0.8	0.6	1.1	0.7
**Variance**	412.6	2.6	2.1	0.6	0.3	1.2	0.5

**Table 3:** Descriptive Statistics of Feta cheese consumption data for adults 20–65 years old according to the Hellenic National Nutrition & Health Survey (HNNHS)**.**

	Consumption Per Capita Per Day (g)
Mean	50.3
Standard Error	1.0
Median	39.0
Mode	39.0
Fifth Percentile	20
50th Percentile	39
95th Percentile	100
Standard Deviation	36.2

**Table 4:** Nutrient daily intake (kcal or g) from prepacked feta cheese consumption marketed in the Greek market as affected by product content and daily consumption by Greek consumers.

	Feta Daily Consumption (g)		
Content (g) in Prepacked Feta Products	Fifth Percentile	50th Percentile	95th Percentile
	**Energy (kcal)**		
Fifth Percentile	51	99	255
50th Percentile	55	108	276
95th Percentile	62	122	312
	**Total Fat (g)**		
Fifth Percentile	4.2	8.2	21.0
50th Percentile	4.6	9.0	23.0
95th Percentile	5.4	10.5	26.8
	**Saturated Fat (g)**		
Fifth Percentile	2.7	5.2	13.4
50th Percentile	3.2	6.2	16.0
95th Percentile	3.7	7.3	18.7
	**Carbohydrates (g)**		
Fifth Percentile	0.0	0.0	0.0
50th Percentile	0.1	0.3	0.7
95th Percentile	0.5	1.0	2.5
	**Sugars (g)**		
Fifth Percentile	0.0	0.0	0.0
50th Percentile	0.1	0.2	0.5
95th Percentile	0.2	0.4	1.1
	**Salt (g)**		
Fifth Percentile	0.2	0.5	1.2
50th Percentile	0.5	1.0	2.5
95th Percentile	0.7	1.3	3.3
	**Protein (g)**		
Fifth Percentile	3.0	5.8	15.0
50th Percentile	3.3	6.4	16.5
95th Percentile	3.6	7.1	18.1

**Table 5:** Percentages of feta cheese products that met the respective criteria of five chosen nutrient profile models.

Nutrient Profile Models	(I)	(II)	(III)	(IV)	(V)
Results	% of Products passing or failing model’s criteria				
Pass				100 *	5
Fail	100	100	100		84
Not Applicable					11

* The score varied between 17 and 23 points.
